# Label-free data standardization for clinical metabolomics

**DOI:** 10.1186/s13040-017-0132-x

**Published:** 2017-02-28

**Authors:** Petr G. Lokhov, Dmitri L. Maslov, Oleg N. Kharibin, Elena E. Balashova, Alexander I. Archakov

**Affiliations:** Institute of Biomedical Chemistry, Pogodinskaya st.10, 119121 Moscow, Russia

**Keywords:** Data standardization, Mass spectrometry, Blood plasma, Clinical metabolomics

## Abstract

**Background:**

In metabolomics, thousands of substances can be detected in a single assay. This capacity motivates the development of metabolomics testing, which is currently a very promising option for improving laboratory diagnostics. However, the simultaneous measurement of an enormous number of substances leads to metabolomics data often representing concentrations only in conditional units, while laboratory diagnostics generally require actual concentrations. To convert metabolomics data to actual concentrations, calibration curves need to be generated for each substance, and this process represents a significant challenge due to the number of substances that are present in the metabolomics data. To overcome this limitation, a label-free standardization algorithm for metabolomics data is required.

**Results:**

It was discovered that blood plasma has a set of stable internal standards. The appropriate usage of these newly discovered internal standards provides a background for the label-free standardization of metabolomics data that underlies the SantaOmics (Standardization algorithm for nonlinearly transformed arrays in Omics) algorithm. In this study, using the knee point, it was shown that the metabolomics data can be converted by SantaOmics into a standardized scale that can substitute actual concentration measurements, thus making the metabolomics data directly comparable with each other as well as with reference data presented in the same scale.

**Conclusion:**

The developed algorithm sufficiently facilitates the usage of metabolomics data in laboratory diagnostics.

**Electronic supplementary material:**

The online version of this article (doi:10.1186/s13040-017-0132-x) contains supplementary material, which is available to authorized users.

## Background

In metabolomics-related methods, a large number of substances can be detected in a single sample [1]. In the case of bodily fluid samples, this capacity provides great potential for diagnostics [2, 3] that have been confirmed in numerous studies [1, 4]. However, omics sciences, including metabolomics and proteomics, generally do not express the concentrations of the detected substances in actual values. For example, in the mass spectrometric analysis of the blood plasma metabolome, the mass peak intensities reflect the substance concentrations in the plasma. These mass peak intensities are expressed in conditional units that are instrument-dependent: the type, model, settings, operating state, etc. of the mass spectrometer directly fluctuate the peak intensity. Therefore, fingerprints, patterns, barcodes, signatures, etc. [5], which have been successfully used in numerous metabolomics-related ‘case–control’ studies, are unsuitable for laboratory diagnostics. In ‘case–control’ studies, the samples are typically analyzed under the same conditions and compared with each other. This avoids the need to determine the actual concentrations of the analyzed substances. However, to convert mass peak intensities to actual concentrations, which is mandatory for medical purposes, calibration curves need to be generated for each substance. This process represents a significant challenge due to the number of substances detected in the metabolomics technologies and the limited number of commercially available chemical standards required to build calibration curves.

The goal of this study was to overcome the aforementioned problem facing the development of metabolomics tests. To this end, an algorithm that converts metabolomics data of blood plasma into a standardized scale and directly allows comparison with other data as well as with reference data was developed. This type of conversion allows the measurement of thousands of substances in a single assay without using calibration curves that may sufficiently facilitate the usage of metabolomics data in laboratory diagnostics. This algorithm was named SantaOmics (Standardization algorithm for nonlinearly transformed arrays in Omics).

## Methods

### Blood plasma sample preparation

Venous blood was collected from three volunteers (33-, 24-, and 23-year-old males) into EDTA Vacutainer plasma tubes (BD, USA). Blood samples were processed according to the manufacturer’s instructions. The resultant blood plasma was stored at −80 °C until analysis. The analyzed samples were subjected to one freeze/thaw cycle. Plasma (10 μL) was mixed with 10 μL of water (LiChrosolv; Merck KGaA, Darmstadt, Germany) and 80 μL of methanol (Fluka, Munich, Germany). Next, after incubation at room temperature for 15 min, the samples were centrifuged at 13,000 × *g* (MiniSpin plus centrifuge; Eppendorf AG, Hamburg, Germany) for 10 min. Supernatants were then transferred to clean plastic Eppendorf tubes, and fifty volumes of methanol containing 0.1% formic acid (Fluka) was added to each tube. The resultant solution was subjected to mass spectrometric analysis. The study design was approved by the relevant ethical review committee.

To confirm that the volunteers did not have a sufficiently distorted blood plasma composition, basic biochemical and blood parameters of these volunteers were measured using routine automatic analyzers. Most of the parameter values fit within normal ranges known in the field of clinical laboratory practice (see Additional file 1: Table S1).

### Mass spectrometry analysis

Samples were analyzed by direct infusion mass spectrometry with a maXis hybrid quadrupole time-of-flight mass spectrometer (Bruker Daltonics, Billerica, MA, USA), a micrOTOF-Q hybrid quadrupole time-of-flight mass spectrometer (Bruker Daltonics, Billerica, MA, USA), an OrbiTrap Elite mass spectrometer (Thermo Scientific, USA), a Fourier transform ion cyclotron resonance mass spectrometer (Apex Ultra, Bruker Daltonics, USA), and with an IFunnel Q-ToF mass spectrometer 6550 (Agilent Technologies, USA) equipped with an electrospray ion sources. Details are described in the supplementary material [see Additional file 2]. The resultant metabolite ion masses were pooled and processed using Matlab version R2010a (MathWorks, Natick, MA, USA). This and all other calculations were performed using Matlab software.

### Mass spectra standardization by the SantaOmics algorithm

**Fig 1:**
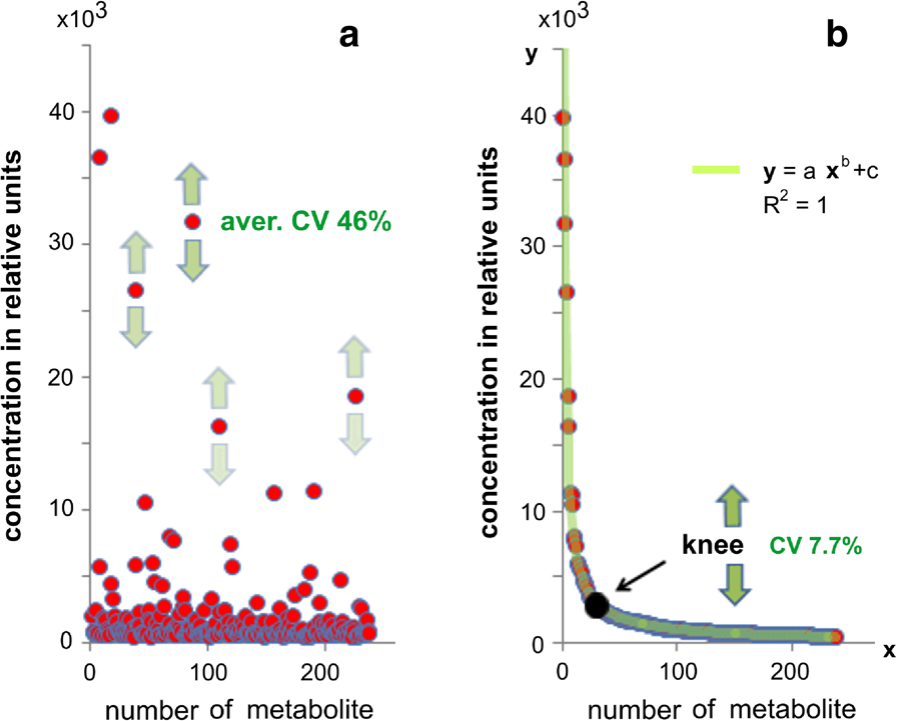
The concept for label-free standardization of metabolomics data. The actual concentrations of substances in the blood plasma sample cover several orders of magnitude (**a**). When the substances are plotted according to their decreasing peak intensity, their distribution obeys the *power* function (**b**). This function helps to find the knee point for this distribution, which is defined by the assemblage of substances that makes it tolerant to concentration fluctuations of separate substances. Moreover, the knee point is independent of the LOD of the method, which may affect only the lower tail of the curve presented on the plot. While the substances demonstrate a high concentration variation (e.g., average CV for biological variation of the blood plasma metabolites is equal to 46% [6]), the knee point demonstrates a relatively low CV (7.7%). So, since the knee point is stable, it may be used as a internal standard that is represented in all blood plasma samples. This artwork was prepared as an example using actual mass spectrometric data for human blood plasma metabolites in the *m/z* range of 225–275 (see details in Methods). R^2^, coefficient of determination for the substances arranged according to their decreasing peak intensities and the *power* function

A fragment width of *m/z* 50 was selected as the start of the mass spectrum (i.e., at the edge with the lowest *m/z* values). The mass spectrometric peaks located inside the fragment were arranged according to their decreasing mass peak intensities. The curve approximating the intensity values was built using the *fit* function (here and below all mentioned mathematical functions are from Matlab software) with the *power* equation (y = *ax* ^*b*^ *+ c*) as the approximation type. The knee point of this curve was established by finding the first and second derivatives (the source code is presented in the data repository). The knee point determined the normalization value (see Fig. 1) for the *m/z* value in the middle of the selected fragment. Iteratively, until the entire mass spectrum was processed, the fragment was shifted by *m/z* 1 and all calculations were repeated. The calculated normalization points were approximated by the curve (called the normalization curve) using the *fit* function (*smoothing splaine* as the approximation type). In order to obtain a mass spectrum in a dimensionless instrument-independent scale, the intensity of each mass peak was divided by the value of the normalization curve in the *m/z* point of the corresponding peak.

### Test for knee point stability

The mass spectrometric data for the human blood plasma metabolome at *m/z* 225–275 was taken as an example of the omics data, and the mass peak intensities were fluctuated (by iterations of 10) by random noise (by the use of the function *normrnd*) in order to provide a peak intensity CV equal to 46%, the average CV for biological variation of the metabolites in blood plasma [6]. Simultaneously, the CV for the knee point was measured with the aim to estimate its stability.

### Tests for the SantaOmics algorithm

The first test was related to the capacity of the SantaOmics algorithm to correct the undesired variability in the mass spectra. The mass list from the mass spectrum of sample #3, obtained at a ‘low range’ mode of detection, was sufficiently distorted. The mass peak intensities were multiplied (10×), linearly increased (from 1× in the low *m/z* area, to 10× in the high *m/z* area), or nonlinearly distorted using the Gaussian function (intensities in the low and high *m/z* areas were decreased (1/4), while the intensities in the center of the mass spectrum were increased (4×)). So, all possible types of variability were imitated by the selected types of distortions. The distorted mass lists were standardized by the SantaOmics algorithm and then were compared with the standardized nondistorted mass list of sample #3 by means of calculating the R^2^ value for linear approximation of the peak intensities and the correlation coefficient (r).

The second test was related to the capacity of the SantaOmics algorithm to standardize sufficiently divergent data obtained from the same instrument (intra-instrumental experiment). Such divergence may be a result of the variability, which appears when different options are used for measurement. For this aim, the mass spectra obtained by maXis in different ranges of detection (‘wide range’ and ‘high range’) were standardized. The overlapping areas of these mass spectra were used to estimate their identity (by calculating R^2^ and r).

The third test, which demonstrated the capacity of the algorithm to convert mass spectra to the same scale, was performed using mass spectra from different mass spectrometers (inter-instrumental experiment with maXis, micrOTOF-Q, OrbiTrap Elite, Apex Ultra, and IFunnel Q-ToF mass spectrometers, thus providing data for mass spectrometers from the same manufacturer and same design, as well as from different manufacturers and different designs). The mass spectra were overlapped in order to visually compare them in terms of the quality of scaling. Additionally, Spearman correlation coefficient determination and Passing Bablok analysis were performed to measure the slopes and intercepts in three independent experiments corresponding to blood plasma samples from three different volunteers.

## Results

### Development of the label-free data standardization algorithm for metabolomics data

In order to develop the label-free standardization algorithm, the characteristics of the plasma samples required to provide a background for the stable internal standards were determined. Generally, blood plasma samples contain substances with concentrations covering several orders of magnitude (Fig. 1a). If the substances are plotted according to their decreasing peak intensity (a measure of concentration), they will be arranged in a smooth line, which can be easily approximated with the *power* function. The coefficient of determination (R^2^) value for this approximation will be approximately 1 (Fig. 1b). An easily detectable characteristic of this function is the knee point, which corresponds to the maximum curvature of the line built by this function.

To confirm hypothesis that this knee point can be used as an internal standard, the stability of this point was tested. From the previously published data it was known that the blood plasma metabolites demonstrate biological variation, with an average coefficient of variation (CV) equal to 46% (Fig. 1a) [6]. In a model experiment, the metabolite intensities were varied so that their CV was equal to 46%. Such a CV of the metabolites was reflected in the CV of the knee point, which was equal to only 7.7%. Therefore, despite that the metabolites demonstrate a very high CV, it was observed that the knee point is relatively stable. So, this simple model demonstrated that blood plasma samples have a stable internal standard. The knee point was characterized to be independent of the concentrations of separate substances, due to its position being defined by the assemblage of substances. In addition, the knee point was independent of the limit of detection (LOD) of the measurement method (Fig. 1b). Thus, the appropriate usage of this newly discovered internal standard provides a background for the label-free standardization of metabolomics data that underlies the SantaOmics algorithm.

### Application of SantaOmics to blood plasma metabolomics data

**Fig. 2:**
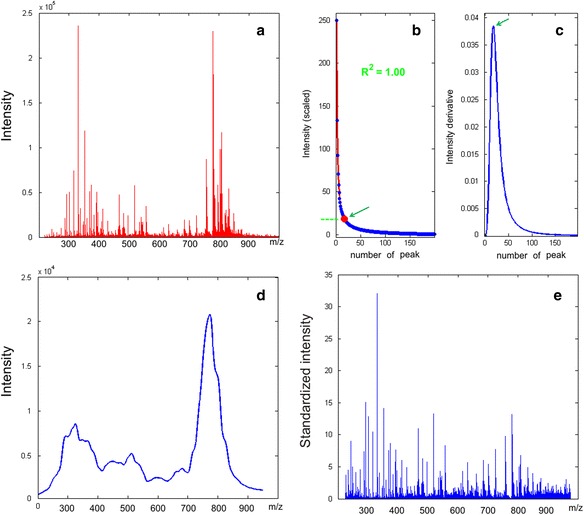
Typical mass spectra of human blood plasma metabolites standardized according to the SantaOmics algorithm. **a** The initial mass spectrum of human plasma metabolites. The mass spectrum was obtained after the direct infusion of a blood plasma sample into an electrospray ion source of a hybrid quadrupole time-of-flight mass spectrometer (maXis, Bruker Daltonics). **b** Detection of the normalization value for a particular mass (*m/z* 225) in the mass spectrum. The substances from the selected range (*m/z* 225 ± 50) of the mass spectrum are plotted according to their decreasing peak intensity. The place of maximum curvature of the curve (knee point), which approximates the range of intensities, corresponds to the normalization value (*depicted by the arrow*). **c** Maximum curvature detection by intensity derivative calculations. The derivative maximum (*depicted by the arrow*) corresponds to the knee point, which indicates the normalization value on the y-axis of plot B. **d** Normalization curve that was built by approximation of the normalization values calculated over the entire range of the mass spectrum. **e** Standardized mass spectrum that was obtained by dividing the peak intensities of the initial mass spectrum by the normalization curve

The mass spectrometric analysis of human plasma samples resulted in the detection of ~17 thousands metabolite ions. Figure 2a illustrates a typical mass spectrum of human blood plasma.

Figure 2b and c shows the plots of substances arranged according to their decreasing mass peak intensities. From these plots, the knee point was determined to be the point of maximum curvature of the function approximating these intensities. A set of such knees was calculated for each part (with *m/z* 1 shift) of the mass spectrum and was used to build a normalization curve for the entire mass spectrum (Fig. 2d). In order to obtain a mass spectrum in the standardized form, the intensity of each mass peak was divided by the value of the normalization curve at the *m/z* point of the corresponding peak. Figure 2e depicts the standardized mass spectrum, and the peak intensities are presented in an instrument-independent scale.

### Application of SantaOmics to distorted metabolomics data

**Fig. 3:**
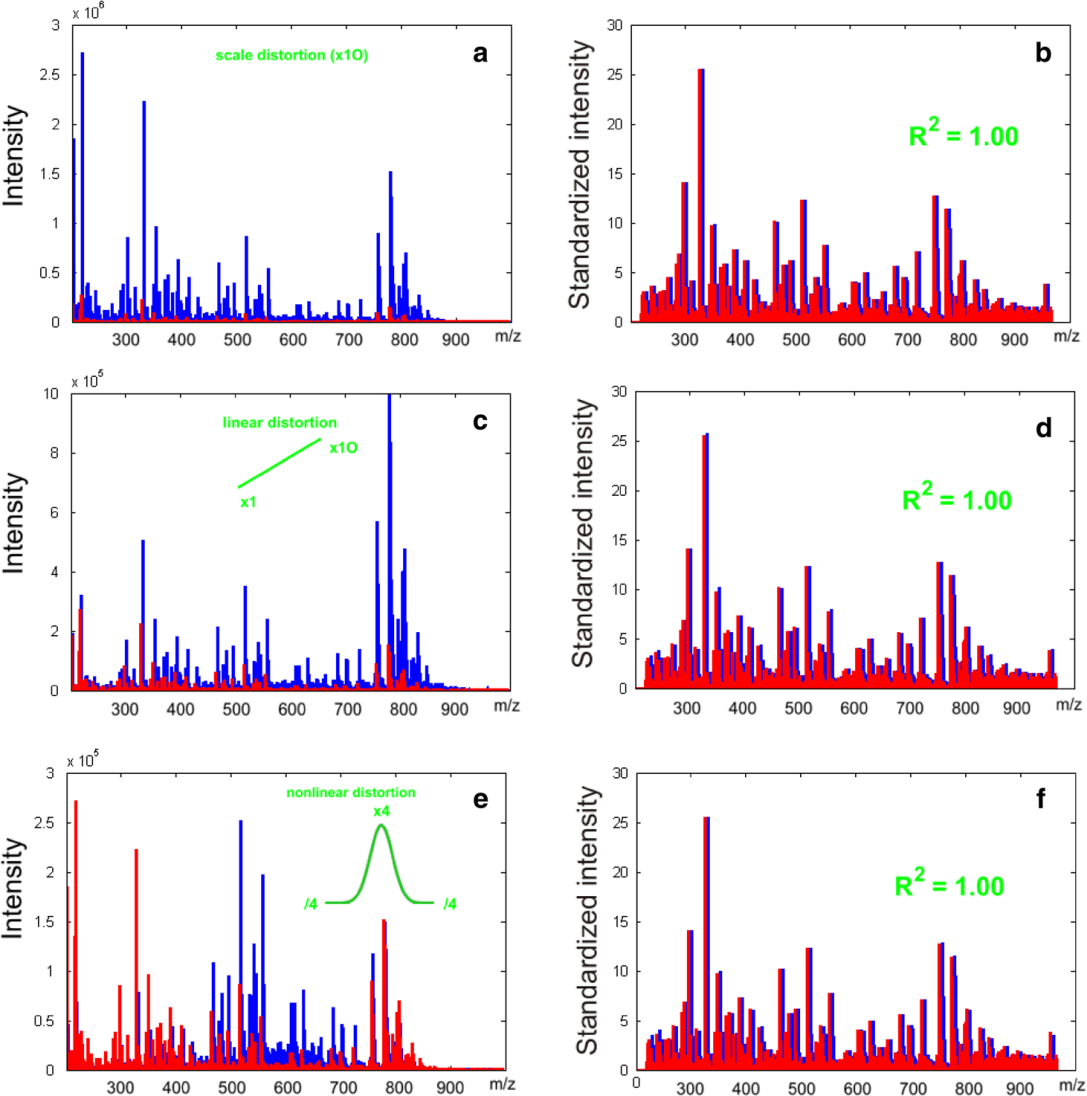
The test results of the SantaOmics algorithm. The mass peaks were extensively distorted in different ways, and the SantaOmics algorithm was applied to standardize the distorted mass spectra. The initial and distorted by multiplication (10×) mass spectra before (**a**) and after (**b**) standardization. Initial and linearly distorted (right corner is suppressed, left corner is powered) mass spectra before (**c**) and after (**d**) standardization. Initial and nonlinearly distorted (right and left corner are suppressed, center of spectrum powered) mass spectra before (**e**) and after (**f**) standardization. R^2^, coefficient of determination for linear approximation of the data; the value equal to 1 confirmed that the SantaOmics algorithm is capable of correcting extensive distortions in the mass spectra

To confirm the capacity of the SantaOmics algorithm to standardize the metabolomics data, the algorithm was applied to crudely distorted mass spectra of the blood plasma metabolome. Figure 3 shows the initial and linearly distorted as well as nonlinearly distorted mass spectra before and after standardization by the SantaOmics algorithm. Notably, such distortions cover all possible undesired variations in mass spectral data that may be met in reality (e.g., signal drift, ion suppression of signal, different signal strengths in different parts of the mass spectrum, etc.). The quality of standardization was estimated by linear approximation for the corresponding peak intensities. R^2^ was equal to 1, the slope was approximately 1, and the intercept was approximately zero for all cases, allowing that the initial and distorted mass spectra after standardization become equal and in the same scale. Therefore, any possible distortions presented in a mass spectrum will be removed by the standardization procedure. So, metabolomics data can be standardized and then compared with reference data as well as with diagnostic signatures expressed in the same scale, thus excluding the requirement of using actual concentrations.

### Application of SantaOmics to divergent intra-instrumental metabolomics data

**Fig. 4:**
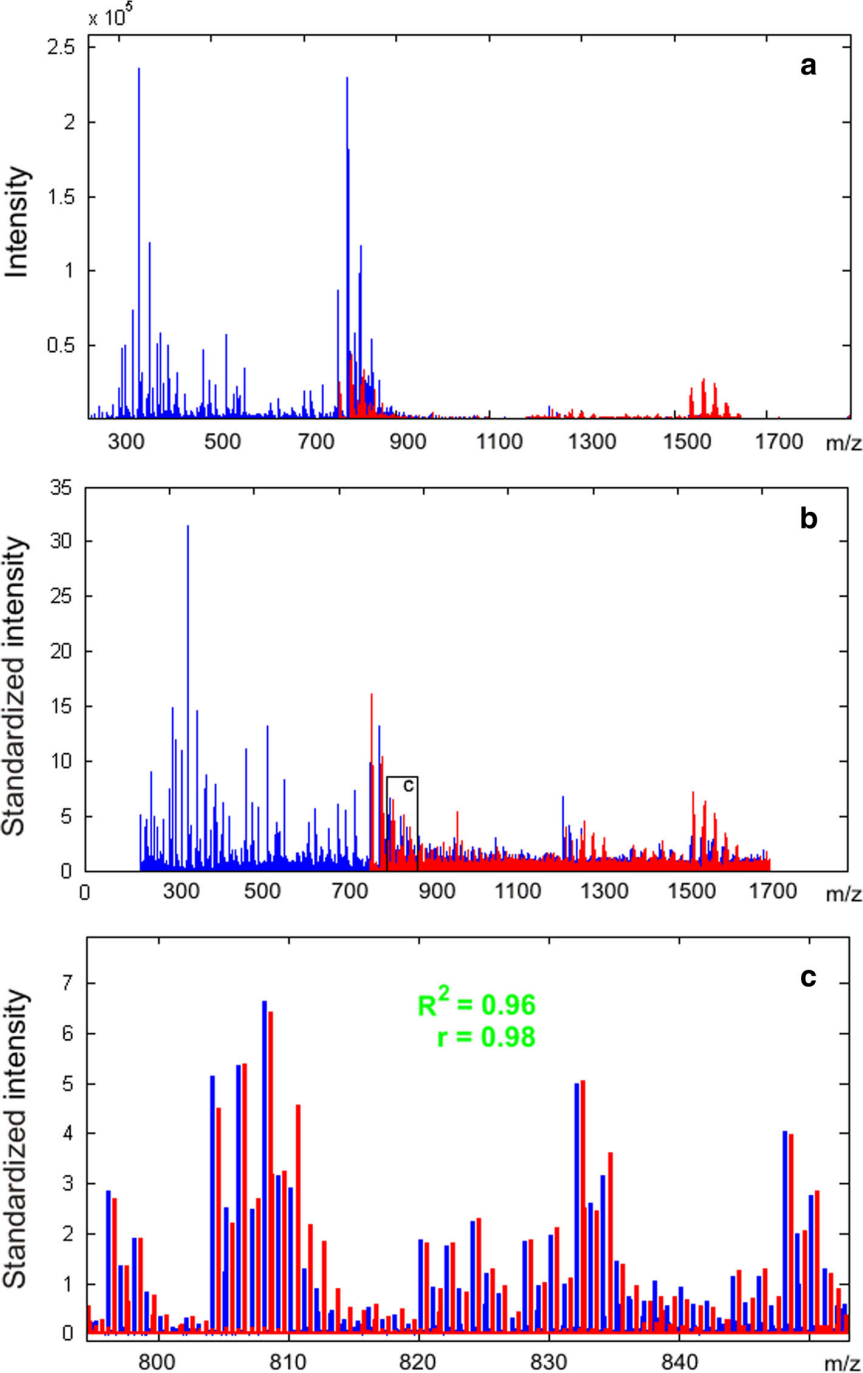
Mass spectra of the same blood plasma sample obtained at different ranges of mass detection before (**a**) and after (**b**) standardization, according to the SantaOmics algorithm. The overlapping area of the standardized mass spectra (**c**) demonstrated the similarity in peak intensities. R^2^, coefficient of determination for linear approximation of the data calculated for peak intensities; r, correlation coefficient

The capacity of the algorithm to standardize divergent metabolomics data obtained using different instrument measurement options was tested. The ‘wide’ and ‘high’ ranges of detection, which are characterized by sufficiently divergent measurement options, were used for mass spectral analysis of the same sample. As a result, two sufficiently different mass spectra were obtained, which were then standardized according to the SantaOmics algorithm. The overlapping areas of the standardized mass spectra were used to calculate the correlation coefficient and R^2^, which were equal to 0.98 and 0.96, respectively (Fig. 4). This test demonstrated that even if sufficiently divergent measurement options were used to obtain the metabolomics data, these data still could be considered as qualitatively standardized scale after applying the algorithm.

### Application of SantaOmics to inter-instrumental metabolomics data

**Fig. 5:**
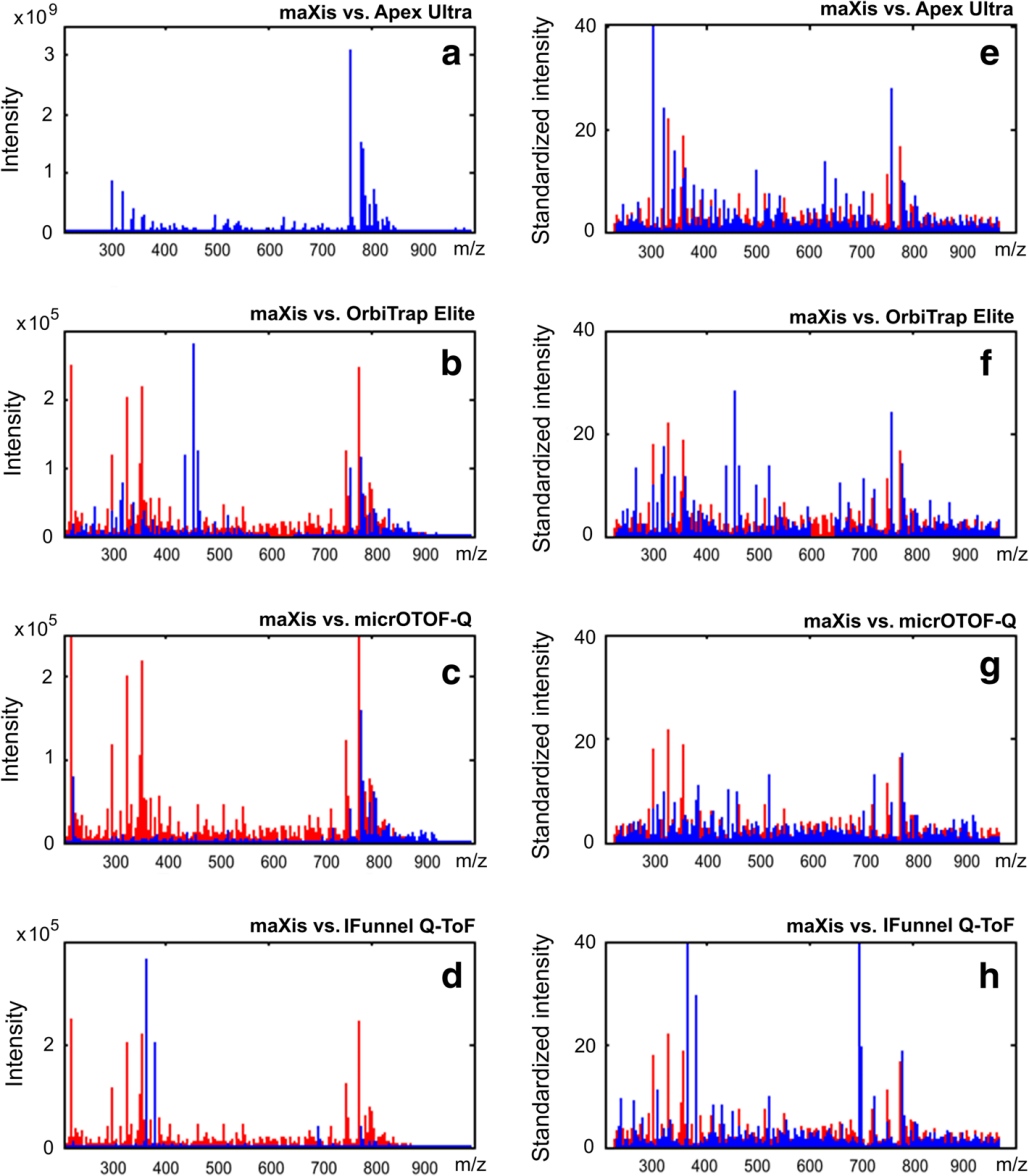
Mass spectra standardization by the SantaOmics algorithm in the inter-instrumental experiment. **a-d** Overlapped mass spectra obtained by maXis and other mass spectrometers before standardization. **e-h** The same mass spectra after standardization

The most difficult examination for the SantaOmics algorithm was standardization of the metabolomics data from different instruments. To this end, the algorithm was applied to the mass spectra of the blood plasma metabolome, which were obtained by different mass spectrometers with different designs as well as options used for mass measurement. Figure 5 visually confirms that the standardized mass spectra are presented in the same scale. The objective equivalence of scales was confirmed by inter-instrumental Passing Bablok analysis (Table 1) of the standardized mass spectra, as shown by the values of the slopes (0.84–1.12) and intercepts, as well as by the Spearman correlation coefficients (0.27–0.73). The same values for hybrid quadrupole time-of-flight mass spectrometers, i.e., for mass spectrometers with the same design, were expectedly better (slope, 0.88–1.09; Spearman correlation coefficients, 0.67–0.73) than for the mass spectrometers with different designs. These data related to the inter-instrumental reproducibility of the method are comparable with data reported for microarrays, i.e., for technology that measures actual concentrations [7].

**Table 1:** Averaged data for Passing Bablok analysis and Spearman correlation for mass spectra of the same biosamples after normalization according to the SantaOmics algorithm

	maXis	Apex Ultra	OrbiTrap Elite	micrOTOF-Q	IFunnel Q-ToF
maXis		0.46 ± 0.08	0.56 ± 0.09	0.70 ± 0.07	0.73 ± 0.04
Apex Ultra	1.01 ± 0.14 0.01 ± 0.01	–	0.68 ± 0.07	0.50 ± 0.07	0.27 ± 0.06
OrbiTrap Elite	1.10 ± 0.22 0.05 ± 0.06	1.12 ± 0.22 0.021 ± 0.04	–	0.58 ± 0.12	0.34 ± 0.10
micrOTOF-Q	0.88 ± 0.16 −0.15 ± 0.09	0.91 ± 0.31 −0.19 ± 0.16	0.84 ± 0.13 −0.19 ± 0.06	–	0.67 ± 0.10
IFunnel Q-ToF	0.99 ± 0.22 0.05 ± 0.03	0.98 ± 0.30 0.03 ± 0.05	0.96 ± 0.39 0.00 ± 0.05	1.09 ± 0.24 0.25 ± 0.03	–

All data are presented as the mean ± standard deviation for three independent experiments (i.e., for samples from three volunteers). Individual data points are presented in Additional file 3: Tables S2-S10. The slopes and intercepts (just below the slope values) for Passing Bablok analysis are presented in the lower left part of the table. The Spearman correlation values are presented in the upper right part of the table

## Discussion

The SantaOmics algorithm, which standardizes metabolomics data and represents them in a standardized scale as a valid substitution of actual concentration measurements, was developed. The discovered stability inside the biosamples was the basis for this algorithm. It was found that the point of maximum curvature (knee) of the curve approximating the concentrations arranged in descending order for the substances presented in a blood plasma sample is stable. Variations in the concentrations of highly abundant substances (corresponding to the high tail of the curve in Fig. 1b) as well as of lowly abundant substances have no effect on the knee point. The concentrations of any separate substances also do not have an effect on the knee point because their position is defined by sets of substances. Because lowly abundant substances do not have an effect on the knee point, its position generally is not dependent on the LOD, thus making the algorithm applicable for different instruments. The basis for such robustness of the knee point was demonstrated in a simple simulation experiment (Fig. 1).

The characteristic nature of the knee point for blood plasma samples and its stability have a fundamental basis. Any biological species is characterized by a genetically defined molecular composition, which distinguishes this species from others and makes it similar to other individuals of the same biological species. The stability of molecular composition is supported by homeostasis. Namely, the molecular composition defines the knee point because it is independent of the concentrations of separate substances. Therefore, the knee point may be considered as a stable and ubiquitous internal standard, which is present in all blood plasma samples and can be found in metabolomics data. This unique internal standard is independent of the type and strength of distortion of the data and allows for label-free standardization of the metabolomics data. This method was demonstrated on mass spectrometric metabolomics data. Dividing the mass peak intensities by knee point values corresponding to *m/z* values of peaks converts the entire mass spectrum into a standardized instrument-independent scale. Consequently, the most applicable use of such standardization in medicine is the capacity to compare mass spectra obtained in different laboratories and with reference data presented using the same standardized scale for the same type of biosamples.

Calculation of the correlation and slope for the linear approximation is a common way to estimate the reproducibility of inter-instrumental array data to which standardized metabolomics data can be related. Previously, such information for metabolomics data could not be obtained because SantaOmics is the first algorithm to make the comparison of inter-instrumental metabolomics data reasonable. Thus, to estimate the correlation and slope values, they can be compared with the same parameters for microarrays. According to data from the MicroArray Quality Control consortium, the comparison of inter-platform microarray data demonstrates a slope of 0.41–2.44 and a correlation of 0.691–0.933 [7]. Therefore, metabolomics arrays, representing standardized mass spectrometric data, demonstrate at least the same inter-platform reproducibility as microarrays, which provide results in actual concentrations and some of which are already accepted for clinical application. The main and most far-reaching conclusion from these results is that metabolomics data already are beyond the limitation of usage only in ‘case–control’ studies and that metabolomics data will soon become suitable for clinical laboratory protocols.

## Conclusions

Using metabolomics, the measurement of almost all low molecular weight substances present in bodily fluids in a single run will have many applications in health care services. Label-free standardization of the test output simplifies data interpretation and allows the biomedical test requirements to be met. The diverse metabolomics signatures that already have been found, as well as will be found in the near future, cover a broad range of tasks for which metabolomics testing can be developed, including disease diagnostics, risk assessment, estimation of biological parameters (e.g., biological age, pregnancy, resistance to stress, fertility, potential longevity, etc.), and biochemical high-throughput screening. All these tests can be performed by applying different signatures to the standardized mass spectra of the blood plasma samples.

## Additional files

Additional file 1: Table S1. Biochemical and blood parameters of the volunteers. (PDF 177 kb)
Additional file 2: Protocols for mass spectrometry analysis. (PDF 81 kb)
Additional file 3: Tables S2-S10. Passing Bablok analysis and Spearman correlation between the different mass spectra after normalization according to the SantaOmics algorithm. (PDF 55 kb)

## Abbreviations

CV: Coefficient of variation
LOD: Limit of detection
SantaOmics: Standardization algorithm for nonlinearly transformed arrays in Omics
