# Supplementary material for: Label-free data standardization for clinical metabolomics
Source: BioData Min. 2017 Feb 28;10:10. doi: 10.1186/s13040-017-0132-x (PMC5329969; doi:10.1186/s13040-017-0132-x)
Supplement: Additional file 1: Table S1. — Biochemical and blood parameters of the volunteers. (PDF 177 kb) [file 13040_2017_132_MOESM1_ESM.pdf]

Supplementary Table S1. Biochemical and blood parameters of the volunteers.

	Normal values (for males)	Volunteer #1	Volunteer #2	Volunteer #3
**Age**		33	24	23
**Gender**		male	male	male
**biochemical analysis**				
aspartate transaminase	**0-37 IU/L**	37.9	30.5	47
alanine aminotransferase	**0-42 IU/L**	20	17.8	45.5
gamma glutamyltransferase	**11-50 ME/L**	58.2	15.4	20.7
glutamate dehydrogenase	**0-7 IU/L**	3.29	4.77	7.36
cholinesterase	**5300-12900 IU/L**	8368	6230	10272
alkaline phosphatase	**80-306 IU/L**	160	149	183
leucine aminopeptidase	**21,0-57,6 IU/L**	41.3	32.1	39.7
total bilirubin	**0-17,1 μM/L**	14.2	12.3	26.9
direct bilirubin	**0-4,30 μM/L**	4.29	2.59	9.85
total amylase	**0-220 IU/L**	78.4	56.9	49.9
alpha amylase	**0-115 IU/L**	16	38	20.1
lipase	**0-190 IU/L**	139	71.5	81.8
pancreatic lipase	**0-60 IU/L**	52.3	32.1	35.9
creatinine	**53-115 μM/L**	89.9	82.8	92.1
urea	**1.7-8.3 mM/L**	4.06	5.79	3.3
total protein	**67-87 g/L**	69.6	72.3	69.6
albumin	**35-50 g/L**	46.9	45.2	48.8
uric acid	**200-420 μM/L**	411	413	431
glucose	**4.2-6.4 mM/L**	5.6	4.88	5.01
fructosamine	**0-285 μM/L**	243	255	251
glycated hemoglobin	**4.5-7.5 %**	6.06	5.54	5.88
creatine phosphokinase (CPK)	**0-190 IU/L**	445	185	851
CPK-MM	**0-190 IU/L**	414.4	161.5	819.5
CPK-MB	**0-24 IU/L**	30.6	23.5	31.5
lactic dehydrogenase	**225-450 IU/L**	395	248	384
oxybutyrate dehydrogenase	**72-182 IU/L**	168	120	165
total cholesterol	**2.8-5.2 mM/L**	6.68	4.87	4.25
HDL cholesterol	**>0.91 mM/L**	2.44	2	1.65
LDL cholesterol	**<4.0 mM/L**	4	2.48	1.98
triglycerides	**0.55-2.30 mM/L**	0.53	0.87	1.36
ferrum	**6.6-26.0 mM/L**	17.1	10.5	39.7
calcium	**2.25-2.67 mM/L**	2.55	2.6	2.37
magnesium	**0.7-1.05 mM/L**	0.71	0.97	1.02
inorganic phosphorus	**0.87-1.45 mM/L**	1.38	1.42	1.04
chlorides	**98-106 mM/L**	98.8	100	105
potassium	**3.5-5.1 mM/L**	4.17	3.57	3.56
sodium	**135-145 mM/L**	136.3	138.2	138.3

acid phosphatase	**0-5.4 IU/L**	4.09	3.09	2.52
prostate acid phosphatase	**0-1.7 IU/L**	1.02	0.81	1.12
**hematology panel**				
absolute WBC count (10^9/L)	**4.0-10.0**	6	5.3	5.1
RBC (10^{12}/L)	**4.0-5.7**	5.03	5.16	4.98
hemoglobulin (g/L)	**130-173**	171	152	166
haematocrit (%)	**34.0-49.0**	44.5	43.5	48.4
absolute thrombocyte level (10^9/L)	**100-400**	171	246	238
lymphocytes (%)	**19.0-45.0**	22.5	40	39.3
absolute lymphocyte count (10^9/L)	**1.2-4.0**	1.4	2.1	2
monocytes (%)	**3.0-11.0**	4.4	6.2	7.8
absolute monocyte count (10^9/L)	**0.09-0,60**	0.3	0.3	0.4
granulocytes (%)	**42.0-85.0**	73.1	53.8	52.9
absolute granulocyte count (10^9/L)	**2.0-5.8**	4.3	2.9	2.7
red cell distribution width (%)	**11.5-14.5**	13.7	13.7	13.9
platelet Crit (%)	**0.08-1.00**	0.14	0.12	0.13
mean thrombocyte volume (fL)	**6.0-11.0**	8.3	6	5.6
IgA (g/L)	**0.70-4.00**	2.07	2.27	2.72
IgM (g/L)	**0.40-2.30**	0.94	0.82	1.14
IgG (g/L)	**7.0-16.00**	8.14	11.28	9.97
