# Supplementary material for: Label-free data standardization for clinical metabolomics
Source: BioData Min. 2017 Feb 28;10:10. doi: 10.1186/s13040-017-0132-x (PMC5329969; doi:10.1186/s13040-017-0132-x)
Supplement: Additional file 2: — Protocols for mass spectrometry analysis. (PDF 81 kb) [file 13040_2017_132_MOESM2_ESM.pdf]

Protocols for mass spectrometry analysis

Samples were analyzed by using a maXis hybrid quadrupole time-of-flight mass spectrometer (Bruker Daltonics, Billerica, MA, USA) equipped with an electrospray ionization (ESI) source (nebulized gas, 4 L/min; capillary inlet temperature, 180 °C; capillary voltage, 4.0 kV). The mass spectrometer was set up to prioritize the detection of ions with a mass-to-charge ratio (*m/z*) ranging from 70 to 1000 (‘low range’ mode), from 220 to 1300 (‘wide range’ mode), and from 740 to 1700 (‘high range’ mode), with a mass accuracy of 1–4 parts per million (ppm). Spectra were recorded in the positive ion charge detection mode. Samples were injected into the ESI source using a glass syringe (Hamilton Bonaduz AG, Bonaduz, Switzerland) connected to a syringe injection pump (KD Scientific, Holliston, MA, USA). The flow rate of samples to the ionization source was 180 µL/h. Mass spectra were obtained using DataAnalysis version 3.4 software (Bruker Daltonics) to summarize the 1-min signals. Ion metabolite masses were determined from the mass spectral peaks obtained using the DataAnalysis program. To generate mass lists, the following parameters were used: peak width, 2, 5, and 10; signal-to-noise ratio (S/N), 1, 1, and 1; relative intensity threshold, 0.005%, 0.05%, and 0.05%; absolute intensity threshold, 100, 50, and 100 for ‘low,’ ‘wide,’ and ‘high’ ranges, respectively.

Samples were analyzed by using a micrOTOF-Q hybrid quadrupole time-of-flight mass spectrometer (Bruker Daltonics, Billerica, MA, USA) equipped with an ESI source (nebulized gas, 4 L/min; capillary inlet temperature, 180 °C; capillary voltage, 4.5 kV). The mass spectrometer was set up to prioritize the detection of ions with *m/z* ranging from 80 to 1000 (‘low range’ mode), with a mass accuracy of 2–4 ppm. Spectra were recorded in the positive ion charge detection mode. Samples were injected into the ESI source using a glass syringe (Hamilton Bonaduz AG, Bonaduz, Switzerland) connected to a syringe injection pump (KD Scientific, Holliston, MA, USA). The flow rate of samples to the ionization source was 180 µL/h. Mass spectra were obtained using DataAnalysis version 3.4 software (Bruker Daltonics) to summarize the 1-min signals. Ion metabolite masses were determined from the mass spectral peaks obtained using the DataAnalysis program. To generate mass lists, the following parameters were used: peak width, 2; S/N, 2; relative intensity threshold, 0.01%; absolute intensity threshold, 200.

Samples were analyzed by using an OrbiTrap Elite mass spectrometer (Thermo Scientific, USA) equipped with an ESI source (nebulized gas, 8 L/min, capillary inlet temperature, 270 °C; capillary voltage, 4.2 kV). The mass spectrometer was set up to prioritize the detection of ions with *m/z* ranging from 200 to 1000, with a mass accuracy of 1–5 ppm and a mass resolution of 60,000.

Mass spectra were recorded in the positive ion charge detection mode. Samples were injected into the ESI source using a glass syringe (Hamilton Bonaduz AG, Bonaduz, Switzerland) connected to a syringe injection pump (KD Scientific, Holliston, MA, USA). The flow rate of samples to the ionization source was 300 μL/h. Mass spectra were obtained using Thermo Xcalibur Qual Browser 2.2 (Thermo Fisher Scientific) to summarize the 1-min signals. Ion metabolite masses were determined from the mass spectral peaks obtained using the Xcalibur Qual Browser program. The resultant spectra were obtained by averaging 200 scans.

Samples were analyzed by using a Fourier transform ion cyclotron resonance mass spectrometer (Apex Ultra, Bruker Daltonics, USA) equipped with an ESI source (nebulized gas, 2 L/min; capillary inlet temperature, 220 °C; capillary voltage, 4.1 kV). The mass spectrometer was set up to prioritize the detection of ions with *m/z* ranging from 150 to 1000, with a sub-ppm mass accuracy. Spectra were recorded in the positive ion charge detection mode. Samples were injected into the ESI source using a glass syringe (Hamilton Bonaduz AG, Bonaduz, Switzerland) connected to a syringe injection pump (KD Scientific, Holliston, MA, USA). The flow rate of samples to the ionization source was 180 μL/h. Mass spectra were obtained using DataAnalysis version 3.4 software (Bruker Daltonics) to summarize the 1-min signals. Ion metabolite masses were determined from the mass spectral peaks obtained using the DataAnalysis program. To generate mass lists, the following parameters were used: peak width, 2; S/N, 4; relative intensity threshold, 0.01%; absolute intensity threshold, 100.

Samples were analyzed by using an IFunnel Q-ToF mass spectrometer 6550 (Agilent Technologies, USA) equipped with an ESI source (nebulized gas, 9 L/min; capillary inlet temperature, 220 °C; capillary voltage, 3.9 kV). The mass spectrometer was set up to prioritize the detection of ions with *m/z* ranging from 150 to 1000, with a sub-ppm mass accuracy. Spectra were recorded in the positive ion charge detection mode. Samples were injected into the ESI source using a glass syringe (Hamilton Bonaduz AG, Bonaduz, Switzerland) connected to a syringe injection pump (KD Scientific, Holliston, MA, USA). The flow rate of samples to the ionization source was 600 μL/h. Mass spectra were obtained using LC/MS Data Acquisition for 6200 series TOF and 6500 series Q-TOF (Agilent Technologies, USA) to summarize the 1-min signals. Ion metabolite masses were determined from the mass spectra using the *mspeaks* function and the default options.
