# Supplementary material for: Label-free data standardization for clinical metabolomics
Source: BioData Min. 2017 Feb 28;10:10. doi: 10.1186/s13040-017-0132-x (PMC5329969; doi:10.1186/s13040-017-0132-x)
Supplement: Additional file 3: Tables S2-S10. — Passing Bablok analysis and Spearman correlation between the different mass spectra after normalization according to the SantaOmics algorithm. (PDF 55 kb) [file 13040_2017_132_MOESM3_ESM.pdf]

Passing Bablok analysis and Spearman correlation between the different mass spectra after normalization according to the SantaOmics algorithm.

Supplementary Table S2. Slope for Passing Bablok analysis of sample **#1**.

	maXis	Apex Ultra	OrbiTrap Elite	micrOTOF-Q	IFunnel Q-ToF
maXis		0.87	1.14	1.20	0.87
Apex Ultra	1.14		1.07	1.56	1.05
OrbiTrap Elite	0.87	0.92		1.01	0.72
micrOTOF-Q	0.83	0.63	0.98		0.74
IFunnel Q-ToF	1.14	0.94	1.37	1.33	

Supplementary Table S3. Intercept for Passing Bablok analysis of sample **#1**.

	maXis	Apex Ultra	OrbiTrap Elite	micrOTOF-Q	IFunnel Q-ToF
maXis		0.00	-0.08	0.15	-0.02
Apex Ultra	-0.00		-0.04	0.14	-0.02
OrbiTrap Elite	0.07	0.04		0.26	0.041
micrOTOF-Q	-0.13	-0.09	-0.25		-0.16
IFunnel Q-ToF	0.02	0.02	-0.06	0.22	

Supplementary Table S4. Spearman correlation coefficients for sample **#1**.

	maXis	Apex Ultra	OrbiTrap Elite	micrOTOF-Q	IFunnel Q-ToF
maXis		0.54	0.61	0.75	0.68
Apex Ultra	0.54		0.70	0.50	0.22
OrbiTrap Elite	0.61	0.70		0.54	0.31
micrOTOF-Q	0.75	0.50	0.54		0.70
IFunnel Q-ToF	0.68	0.22	0.31	0.70	

Supplementary Table S5. Slope for Passing Bablok analysis of sample **#2**.

	maXis	Apex Ultra	OrbiTrap Elite	micrOTOF-Q	IFunnel Q-ToF
maXis		1.16	0.75	0.94	0.90
Apex Ultra	0.86		0.73	0.80	0.77
OrbiTrap Elite	1.31	1.35		1.29	1.09
micrOTOF-Q	1.06	1.24	0.77		0.92
IFunnel Q-ToF	1.11	1.29	0.91	1.08	

Supplementary Table S6. Intercept for Passing Bablok analysis of sample **#2**.

	maXis	Apex Ultra	OrbiTrap Elite	micrOTOF-Q	IFunnel Q-ToF
maXis		-0.01	0.01	0.22	-0.07
Apex Ultra	0.01		0.02	0.29	0.01
OrbiTrap Elite	-0.02	-0.02		0.21	-0.04
micrOTOF-Q	-0.24	-0.36	-0.16		-0.25
IFunnel Q-ToF	0.07	-0.01	0.03	0.27	

Supplementary Table S7. Spearman correlation coefficients for sample **#2**.

	maXis	Apex Ultra	OrbiTrap Elite	micrOTOF-Q	IFunnel Q-ToF
maXis		0.43	0.47	0.61	0.77
Apex Ultra	0.43		0.73	0.56	0.24
OrbiTrap Elite	0.47	0.73		0.71	0.26
micrOTOF-Q	0.61	0.56	0.71		0.56
IFunnel Q-ToF	0.77	0.24	0.26	0.56	

Supplementary Table S8. Slope for Passing Bablok analysis of sample **#3**.

	maXis	Apex Ultra	OrbiTrap Elite	micrOTOF-Q	IFunnel Q-ToF
maXis		0.96	0.89	1.34	1.34
Apex Ultra	1.03		0.94	1.17	1.42
OrbiTrap Elite	1.11	1.06		1.33	1.67
micrOTOF-Q	0.75	0.85	0.75		1.17
IFunnel Q-ToF	0.75	0.70	0.60	0.85	

Supplementary Table S9. Intercept for Passing Bablok analysis of sample **#3**.

	maXis	Apex Ultra	OrbiTrap Elite	micrOTOF-Q	IFunnel Q-ToF
maXis		-0.02	-0.07	0.10	-0.07
Apex Ultra	0.02		-0.04	0.11	-0.11
OrbiTrap Elite	0.09	0.05		0.20	-0.02
micrOTOF-Q	-0.08	-0.10	-0.15		-0.31
IFunnel Q-ToF	0.05	0.07	0.01	0.26	

Supplementary Table S10. Spearman correlation coefficients for sample **#3**.

	maXis	Apex Ultra	OrbiTrap Elite	micrOTOF-Q	IFunnel Q-ToF
maXis		0.40	0.61	0.73	0.73
Apex Ultra	0.40		0.60	0.42	0.34
OrbiTrap Elite	0.61	0.60		0.48	0.50
micrOTOF-Q	0.73	0.42	0.48		0.76
IFunnel Q-ToF	0.73	0.34	0.45	0.76	
